# Implications from a Network-Based Topological Analysis of Ubiquitin Unfolding Simulations

**DOI:** 10.1371/journal.pone.0002149

**Published:** 2008-05-14

**Authors:** Arun Krishnan, Alessandro Giuliani, Joseph P. Zbilut, Masaru Tomita

**Affiliations:** 1 Institute for Advanced Biosciences, Keio University, Tsuruoka, Japan; 2 Department of Environment and Health, Istituto Superiore di Sanita, Rome, Italy; 3 Department of Molecular Biophysics and Physiology, Rush University Medical Center, Chicago, Illinois, United States of America; Tel Aviv University, Israel

## Abstract

**Background:**

The architectural organization of protein structures has been the focus of intense research since it can hopefully lead to an understanding of how proteins fold. In earlier works we had attempted to identify the inherent structural organization in proteins through a study of protein topology. We obtained a modular partitioning of protein structures with the modules correlating well with experimental evidence of early folding units or “foldons”. Residues that connect different modules were shown to be those that were protected during the transition phase of folding.

**Methodology/Principal Findings:**

In this work, we follow the topological path of ubiquitin through molecular dynamics unfolding simulations. We observed that the use of recurrence quantification analysis (RQA) could lead to the identification of the transition state during unfolding. Additionally, our earlier contention that the modules uncovered through our graph partitioning approach correlated well with early folding units was vindicated through our simulations. Moreover, residues identified from native structure as connector hubs and which had been shown to be those that were protected during the transition phase of folding were indeed more stable (less flexible) well beyond the transition state. Further analysis of the topological pathway suggests that the all pairs shortest path in a protein is minimized during folding.

**Conclusions:**

We observed that treating a protein native structure as a network by having amino acid residues as nodes and the non-covalent interactions among them as links allows for the rationalization of many aspects of the folding process. The possibility to derive this information directly from 3D structure opens the way to the prediction of important residues in proteins, while the confirmation of the minimization of APSP for folding allows for the establishment of a potentially useful proxy for kinetic optimality in the validation of sequence-structure predictions.

## Introduction

There has been renewed interest in understanding the structural and architectural organization of proteins through a network representation of proteins. The belief is that identifying the guiding organizational principles behind protein structures will lead to uncovering the principles behind protein folding. Ever since Anfinsen's experiment in 1973 [1] proved that all the information for a protein to fold into its three dimensional structure is encoded in its primary sequence, many models have been developed based on a host of theoretical, simulated or experimental techniques [2]. The chief among these are the nucleation-propagation model [3], [4], the nucleation-condensation model [5], the sequential and hierarchical model [6], the collapse model [7] and the modular model [8]–[11]. More recently, a unified model of protein folding that is based on the effective energy surface of a poly peptide chain has been introduced by Wolynes et al. [12] according to which protein folding consists of a progressive organization of ensembles of partially folded structures that arise through multiple routes [12]–[14]. Regardless of the model used, they are all in agreement about the fact that small regions of proteins tend to fold separately and then are aggregated into the final structure for globular proteins by means of stabilizing interactions between the different subunits. In addition, there is broad agreement between models with the predominantly kinetic character of protein folding process: strictly speaking, a minimum energy 3D configuration can be attached to any random amino acid sequence but only those sequences having “kinetically reachable minima” tend to effectively fold in a finite time. This “kinetic first” principle is implicit in the folding funnel paradigm [15].

Graph theory based descriptors of proteins have gained prominence in recent times and have been shown to be ideally suited for studying general topological principles of protein structures [16]. The consideration of proteins as networks by defining the amino acids in the polypeptide chain as the nodes and the noncovalent interactions among them as links allowed us to overcome the need for “artificial” definitions of structural classifications such as motifs, classes, topologies, fold families and superfamilies and to identify some architectural invariants of proteins: at the upper end, we discovered a maximal size for domains at around 275 residues [17] while at the lower end we identified six residue hydrophobic patches or “words” as the smallest unit containing maximal information content as defined by Shannon's entropy [18]. In between these two extremes, we identified modules [19] that were observed to correlate well with early or autonomous folding units (AFUs) or “foldons”. In addition, such a modular partitioning of the protein structure enabled the identification of key residues for protein folding. These residues which play the role of connector hubs, that is, those that connect different modules, were observed to be protected early during the transition phase.

In order to test our two contentions, namely, that the modules identified using our procedure correspond to foldons and that residues that act as connector hubs are protected early in transition phase and hence are more stable, we took recourse to molecular dynamics (MD) simulations by studying the unfolding of ubiquitin. The elucidation of folding kinetics in terms of the different role exerted by amino acid residues on the basis of their topological characterization is of the utmost importance for applicative studies as demonstrated by Stanley and coworkers [20] for the elucidation of amyloid peptide oligomerization process. Dokholyan et al. [21] had shown that topological properties of protein conformations determine their kinetic ability to fold. They observed that there was a difference in the “wiring” between pre- and post transition state (TS) complexes and hypothesized that this could be because of a “specific rewiring” of protein graphs on crossing the free-energy barrier. They also noted that the average minimal distance between any two nodes are smaller for post-transition than pre-transition complexes thereby providing a structurally reliable determinant of the TS. The relevance of topological considerations in folding process can also be determined by the fact that folding is a diffusional process in a non homogeneous medium in which the first passage time (FPT) driving the transition probability is strongly affected by topological properties of the system [15], [22].

In this work, we carried out MD simulations of ubiquitin with a view to analyzing the topological pathway over folding. In other words we try to investigate if the dynamics of protein structure graph descriptors are able to detect the basic features of the entire folding process and not just at the transition state. We identified modular partitions of the protein along the unfolding pathway and analyzed the evolution of the modules as unfolding proceeds. We observed that the modules identified from the native structure using our global graph partitioning approach [19] do indeed correspond to “foldons” and that residues that act as connector hubs are indeed stabilized early during the transition phase. We also observed a significant governing principle of protein folding in terms of the minimization of the sum of the all pairs shortest path (APSP) for all the residues of the protein. APSP can be safely equated to the well known FPT that was demonstrated to play a crucial role in transition probability in a lot of reticular systems [22] and, more specifically, to be deeply involved in allosteric properties of proteins [23].

## Results and Discussion

### Temperature-dependent unfolding simulations for ubiquitin

**Figure 1:**
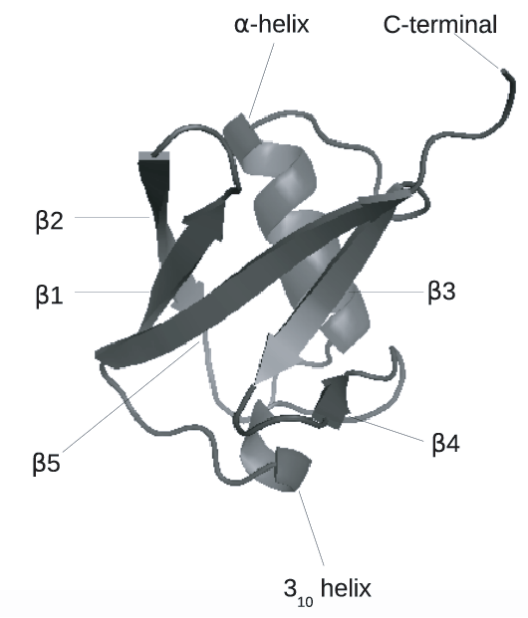
The native structure of ubiquitin (PDB ID: 1UBQ). The secondary structural features are denoted.

Figure 1 shows the native structure of ubiquitin. The native structure consists of five β-strands, an α-helix and a 3_10_-helix. The five β-strands occur between residues 1–8 (β1), 11–17 (β2), 40–45 (β3), 48–50 (β4) and 64–72 (β5). These beta strands form β sheets with the interactions given by β1–β2, β1–β5, β5–β3, and β3–β4 with β1–β2 and β3–β4 forming β hairpins. The two helical structures range from residues 24–34 (α-helix) and 56–59 (3_10_-helix). The core is defined by residues M1, I3, V5, T13, L15, V17 and V26 which all lie in the N-terminal region of the protein.

**Figure 2:**
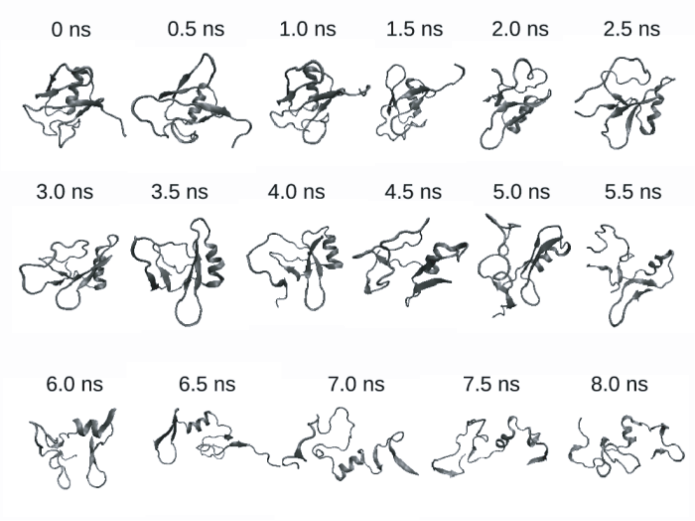
Snapshots of the structure of 1UBQ during unfolding. 500 ps (0.5 ns) snapshots are shown here.

**Figure 3:**
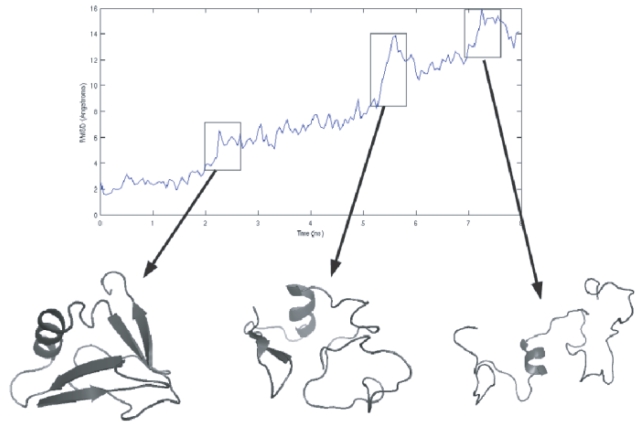
The plot shows the evolution of the RMSD of the backbone C_ atoms from the energy minimized structure of ubiquitin. Three critical phases are discernible. The first phase at ≈2.5 ns involves the breaking of the β_1_–β_5_ interaction. The second phase occurs at ≈5.5 ns and involves the breaking up of the β_3_–β_4_ hairpin along with the associated secondary structure. The third phase occurs at ≈7 ns and involves the breaking up of the β_1_–β_2_ interaction.

Snapshots of the unfolding pathway taken at 0.5 ns intervals are shown in Figure 2 while Figure 3 shows the time-dependent variation of the root mean squared deviation (RMSD) of the backbone atoms (without hydrogen atoms) of the protein from the energy minimized structure. The variation of RMSD with time shows three distinct phases characterized by sharp increases in the RMSD values. The first phase, which occurs at ≈2.5 ns involves the breaking of the β_1_–β_5_ interaction. The second phase occurs at ≈5.5 ns and involves the breaking up of the β_3_–β_4_ hairpin along with the associated secondary structure. The third phase occurs at ≈7 ns and involves the breaking up of the β_1_–β_2_ interaction. The simulation is in line with previous experimental results which suggest that the secondary and tertiary structural features of the N-terminal end of the protein structure (residues 1–33) are comparatively more rigid as compared to the rest of the protein (residues 34–76). The N-terminal end of the protein contains the protein core as mentioned above and this is probably the reason for the rigidity associated with this segment.

### Transition State (TS) determination

**Figure 4:**
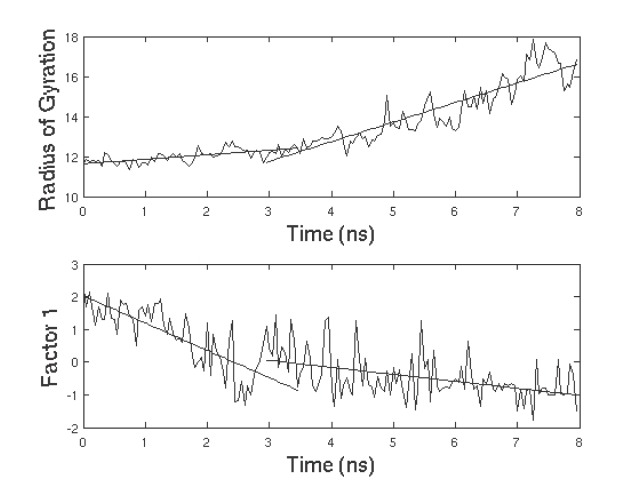
Variation of the Radius of Gyration (RoG) and the first factor obtained from PCA of the RQA quantitative descriptors REC, DET and ENT over time. Top-panel: Variation of RoG with time. Bottom-panel: Variation of Factor-1 with time. In both plots, there are two clearly identified regimes with a marked difference in slope aroud 3 ns indicating a possible location of the TS before this point.

It is well known that ubiquitin exhibits a two state folding behavior and hence the characterization of the transition state is important. The transition state (TS) is a high energy state posited at the peak of the highest energy barrier. Since the TS is kinetically and thermodynamically unstable, it was suggested [24] that the structure of the protein should undergo rapid changes after passing this state. Figure 4 (top panel) shows a plot of the radius of gyration of the protein as unfolding proceeds. There seem to be two distinct phases in the plot identified by their different slopes. The bend in the plot occurs around 2.5–3.0 ns which is probably the location of the TS since it involves a conformation which is near native except for the breaking of the β_1_–β_5_ interaction as mentioned above.

**Figure 5:**
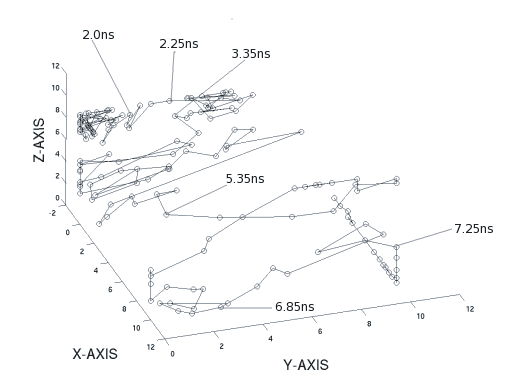
Identification of Transition State: Projection of the RMSD between all pairs of frames onto a 3D cartesian coordinate. The frames were 50 ps apart. The line connecting two two conformations implies sequential evolution in time. The distance between the conformations is proportional to the RMSD distances between the conformations.

However, a more definitive method for obtaining the TS was used by Li et al. [24] who identified the TS by analyzing the RMSD between all pairs of conformations generated during a simulated trajectory. The pairwise RMSD values can be projected onto a 2D or 3D Cartesian space where the distance between conformations correspond to their RMSD from each other. In this work, 50 ps snapshots were chosen from the unfolding trajectory resulting in 160 snapshots or frames. Pairwise RMSDs for each pair of conformations were calculated and were projected onto a 3D Cartesian space (see Materials and Methods) following the work of Dastidar et. al [25]. The projected plot is shown in Figure 5. The line connecting two conformations implies sequential evolution in time. The distance between the conformations is proportional to the RMSD distances between the conformations. Hence, conformations close to each other in the RMSD space will cluster together. However, it must be noted that the coexistence of conformations in a cluster does not imply structural similarity of the conformations but rather that there are no abrupt changes in structure between them. This is at the basis of the so called “chaining effect” [26] which is well known in hierarchical cluster analysis and leads to a grouping of very different objects in the same cluster. Since this is based on the similarity of adjacent conformations, completely unrelated structures can exist in the same cluster [26]. In Figure 5, there are two well-defined clusters. The first cluster extends from 0–2 ns (with an average RMSD of 2.5 °A while the second cluster extends from 2.25–3.25 ns (average RMSD of ≈5.0°A). As mentioned earlier the major structural change takes place at around 2.5 ns and involves the breaking of the β_1_–β_5_ interaction. Hence the period between the two clusters probably indicates the transition between the native structure (the first cluster) and the transient intermediate (the second cluster). This occurs between 2.0 and 2.25 ns and could thus be taken to represent the TS and which also corresponds well with the putative TS inference from the plot of the radius of gyration with unfolding time.

In addition, there are two more regions of large structural changes at ≈5.35 ns and ≈6.9 ns which as we have seen from Figure 3 correspond to the breaking up of the β_3_–β_4_ hairpin and the β_1_–β_2_ interaction respectively. However, these latter transitions occur much later in the simulation and hence, the conformation between 2.0–2.25 ns is a likely candidate for the TS ensemble. Similar to the findings by Dastidar et. al [25], the simulated TS structure is similar to that obtained from experimental -value analysis. It consists of a helix and four beta-sheets including the residual part of the β_3_–β_4_ hairpin. The β_1_–β_5_ interaction is disrupted and this set of interactions possibly provides the energy barrier during the folding process.

### Recurrence Quantification Analysis (RQA) is able to identify the TS

In order to observe the topological variation during the unfolding trajectory, each 50 ps snapshot was analyzed using Recurrence Quantification Analysis (RQA). RQA is a nonlinear technique that is based upon detecting recurrences of similar segments along a given series, as computed by the Euclidean distance below a given radius. As applied to protein graph representation, a recurrence is scored whenever two residues have a distance below 4.5 angstrom, i.e. when they are in contact. This gives rise to a graphical representation named Recurrence Plot (RP) constituted by a square matrix having as rows and columns the residues ordered along the sequence and where each recurrence (contact) is marked by a dot in the RP. The percentage of recurrences over the entire number of possible residue pairs is named REC, the proportion of recurrences occurring along diagonal lines in RP (and thus involving consecutive residues in different portions of the primary structure) is named DET, while the Shannon's information of the length distribution of such diagonal deterministic (DET) lines is called ENT. REC can be considered as a global measure of the density of contacts in the structure [18], DET measures the amount of secondary structure while ENT is related to the dispersion of different line lengths where low values of ENT correspond to the presence of a characteristic length of contact disposition, and high ENT values to the lack of such a characteristic length.

**Figure 6:**
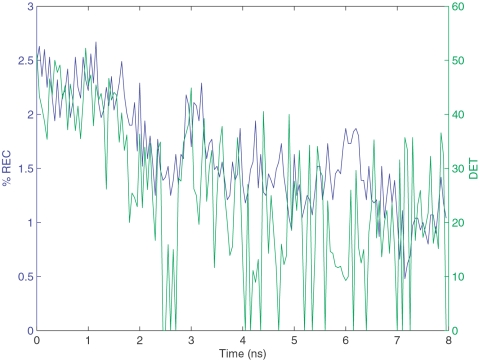
REC and DET over Unfolding.

Figure 6 shows the behavior of REC and DET as unfolding proceeds. As expected, both REC and DET decrease as unfolding progresses. However, when a principal component analysis (PCA) was performed using the values of REC, DET and ENT for all the 160 frames, and the first principal component (Factor1) was plotted vs. unfolding time, it was observed (Figure 4 (bottom panel)) that there were two distinct phases (similar to the plot of radius of gyration vs. time in (Figure 4 (top panel)). The first phase involves a steep decline in the value of the PC followed by a second flattened phase that starts at ≈2.5 ns. This corresponds well to the TS identified in the previous section and hence RQA provides a way to obtain an approximate TS conformation.

The first component (Factor 1) explains 68% of the total variance associated with the data set having as statistical units the 160 time frames and as variables the REC, DET and ENT descriptors. The loadings (Pearson correlation coefficients of original variables with component) are 0.805, 0.862 and −0.807 for REC,DET and ENT respectively. Keeping in mind the definition of the above descriptors, the loading profile suggests that high factor scores imply a relatively high density of contacts (positive correlation with REC), an elevated amount of secondary structure (positive correlation with DET) with a well defined characteristic length (negative correlation with ENT). Thus the decay of Factor1 in the first portion of the graph of (Figure 4 (bottom panel)) (Pearson r between Factor1 and Frame equal to −0.77 for the first 60 Frames) can be interpreted as an index of the progressive elimination of both contacts and secondary structure in the unfolding process (and conversely their formation during the hypothetical folding process).

The lack of any visible link between progression in time and Factor1 during the second phase of the unfolding (Pearson r between Factor1 and Frame equal to −0.24 for the last 60 Frames) is a quantitative proof of the fact that RQA is able to detect the presence of a sharp transition in the process

### Modules obtained using graph partitioning are “foldons”

In an earlier work [19], we had used a well-established, global method for identifying modules in networks. We had demonstrated that these modules correlated well with early folding units or foldons. We analyzed the evolution of the modules as unfolding progressed. This was done by first partitioning each of the 160 frames using our graph partitioning approach. The modules in the first frame were then used as reference sets and the assignment of the residues to their respective modules were noted. For each successive frame, the placement of all pairs of residues was compared against their placement in the first frame. Thus for each frame, a distribution for the placement of all residue pairs was obtained. A two-sample Kolmogorov-Smironov (KS) test was then carried out for each frame vs. every other frame under the null hypothesis that the residue-pair module placement distribution was obtained from the same underlying distribution from both frames. The null hypothesis was tested at a p value of 0.005.

**Figure 7:**
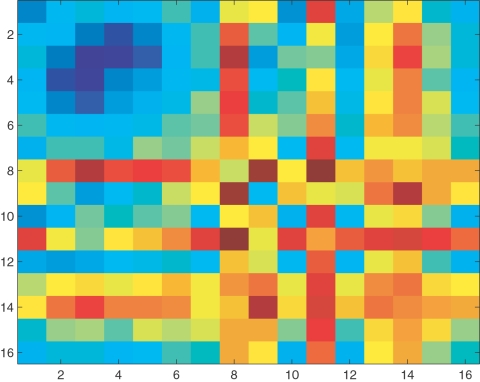
Module composition over unfolding: The plot shows the similarities (differences) in modular composition across the different frames as compared to the composition in the first frame. Each square on the plot represents 0.5 ns. The colors go from blue (similar modular composition) to red (highly dissimilar modular composition). It can be observed that the only region of long-time similarity is in the upper left region which corresponds roughly to about 3 ns, after which the modular compositions become very dissimilar. See text for more details on how the plot was obtained.

The results of the KS test were plotted with a point on the plot representing the rejection of the null hypothesis and a white space representing that the two frames have the same residue-pair module placement distribution. Since frames within 500 ps of each other were found to have similar distributions, squares on the plot with a side = 10 were replaced by averaged values of all the points in the block. Figure 7 shows the result of this averaging procedure where each square represents 0.5 ns and the cooler colors (blue) represent regions where the null hypothesis has been validated. The only predominantly blue region in the figure occurs over the first ≈2.5–3.0 ns indicating that residues that were in the same module in the first frame continue to remain together in succeeding frames until the TS (at 2.5 ns) after which the initially identified modules don't persist. This is in complete accord with our claim in the earlier work that the modules identified were related to early folding units.

### High *|P/z|* valued residues are critical for folding

In addition to our observation that the modules identified using the graph partitioning approach were related to foldons, we had also been able to identify residues critical for folding [19]. The modular decomposition of the protein enabled us to characterize residues based on their intra-module connectivity, *z* and inter-module connectivity or participation coefficient, *P*. Residues that acted as connector hubs between modules, that is, those with high *|P/z|* values were found to be those that were known to be protected early during the transition state from experimental evidence [27].

**Figure 8:**
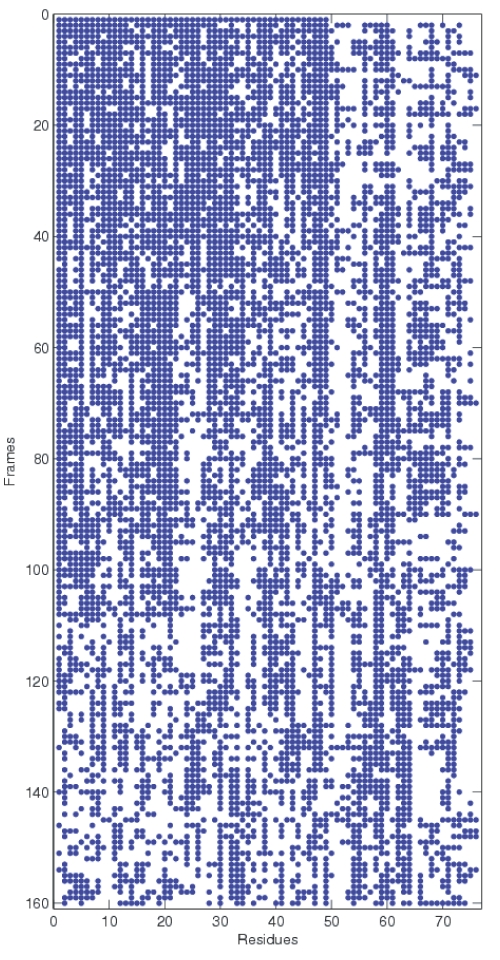
* P/z* over unfolding: The figure shows a plot of the time evolution of the *|P/z|* values of all the residues. The residues have been sorted by their values in the first frame such that the HPZ residues are collected together at the left end of the x-axis. A point on the plot indicates *|P/z|*>0 implying that the corresponding residue has at least one contact outside its own module.

**Figure 9:**
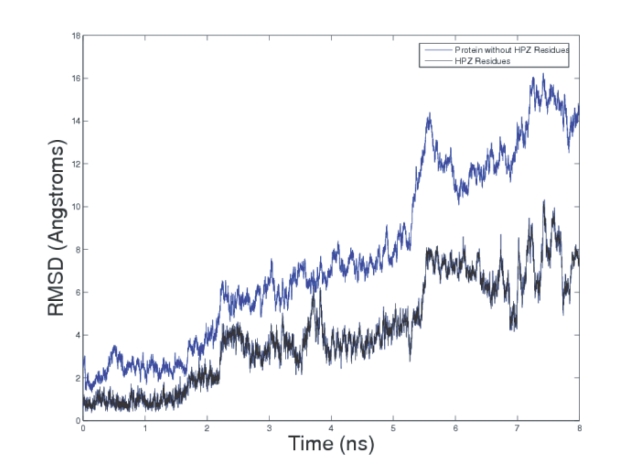
RMSD of Protein without top 5 HPZ residues compared to the RMSD of the top 5 HPZ residues. The HPZ residues have much lower RMSD values than the other residues.

**Figure 10:**
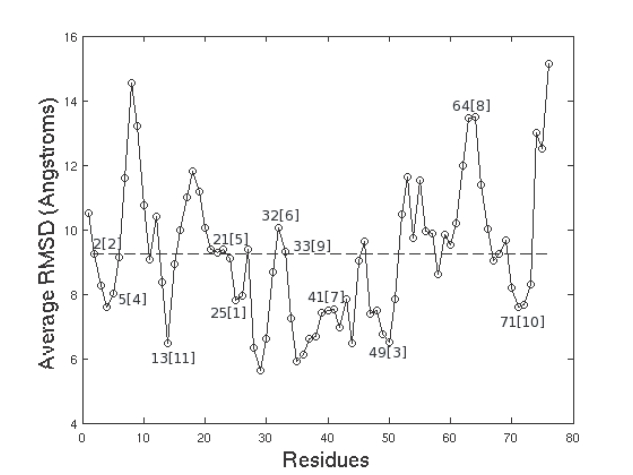
Position of HPZ residues as a function of the average RMSD of the residues. The top 11 HPZ residues are marked with 7/11 of the top HPZ residues lying near the troughs in the plot, indicating their greater stability with respect to the non-HPZ residues.

In order to test our contention that inter-module connector residues were critical for folding, we analyzed the evolution of the high *|P/z|* (HPZ) residues during unfolding. Figure 8 shows a plot of the time evolution of the *|P/z|* values of all the residues. The residues have been sorted by their values in the first frame such that the HPZ residues are collected together at the left end of the x-axis. A point on the plot indicates *|P/z|*>0 implying that the corresponding residue has at least one contact outside its own module. If our earlier contention that HPZ residues were stabilized earlier during the transition phase is true, then we should see them showing persistent inter-module contacts in Figure 8. This is precisely what is observed in the figure. There are two stages of disruption of the residues with inter-module contacts. The residues with lower *|P/z|* (LPZ) residues have their contacts broken at around the 50_th_ frame (≈2.5 ns) while the HPZ valued residues still contain some inter-module contacts till around the 100_th_ frame (≈5 ns.) Thus the HPZ residues are much stabler than the non HPZ residues and hence have lower RMSD values as observed in Figure 9. Indeed, a plot of the average RMSD of the different residues over unfolding (Figure 10) shows that HPZ residues tend to occur around the valleys in the plot. The plot shows the top 11 HPZ residues and as is observed, 7 of the top 11 HPZ occur near the troughs in the plot, strengthening the argument for our original contention that HPZ residues are indeed stabilized early.

**Figure 11:**
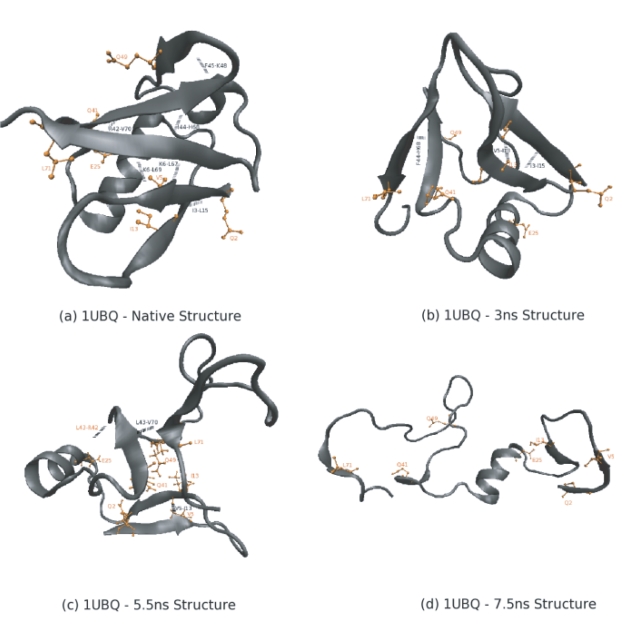
The figure shows the hydrogen bonds in the betasheet of ubiquitin along with the positions of seven of the top 11 HPZ residues (Q2,V5,I13,E25,Q41,Q49,L71). The seven residues are shown in orange. The hydrogen bonds are shown as black dashes with the partners in each hydrogen bond, identified. See text for more details on the comparison of the different structures.

What role do the HPZ residues play in stabilizing the structure? In order to answer this question we compared the native structure of ubiquitin (Figure 11(a)) with structures taken from the three critical phases of unfolding (as discussed earlier) at 3 ns (Figure 11(b)), 5.5 ns (Figure 11(c)) and 7.5 ns (Figure 11(d)). We first identified the hydrogen bonds in the betasheets of the four structures with a cut-off of 3.6°A. The native structure has 6 backbone-backbone (BB) hydrogen bonds (I3-L15, K6-L67, K6-L69,R42-V70,I44-H68 and F45-K48). None of the seven HPZ residues are involved in any hydrogen bonds. At 3 ns, as mentioned earlier, the β_1_–β_5_ interaction is broken and this is shown by the presence of only 3 hydrogen bonds; I3-L15, I44-H68 and the newly formed V5-I13. This last hydrogen bond involves two of the HPZ residues and persists beyond 5.5 ns. However, the other two hydrogen bonds are lost at the 5.5 ns conformation where the β_2_–β_3_ interaction is lost. Two new hydrogen bonds are formed during this stage: L43-V70 and L43-R42 which is a backbone-sidechain (BS) bond. At this point in time, the HPZ residues seem to pack their side-chains towards the center. However, by 7.5 ns, all the hydrogen bonds are lost and the protein is in an unfolded conformation.

Since the only residues that were involved in hydrogen bonding were the V5-I13 pair, we investigated the possibility of the other negatively charged HPZ residues to form salt-bridges. However, none of the HPZ residues were involved in any salt bridges with only three of them (I13,E25 and Q49) being close to residues that formed long-term salt-bridges over the course of the unfolding simulations. The three long-term salt-bridges were formed between K11-N34, K27-N24 and K48-N51 with all three salt-bridges extending through the transition state and well beyond 5 ns during the simulation. Hence, it appears that most of the interactions for the HPZ residues occur through van der Waals contacts, possibly as a result of packing.

### Topological Properties over Unfolding

**Figure 12:**
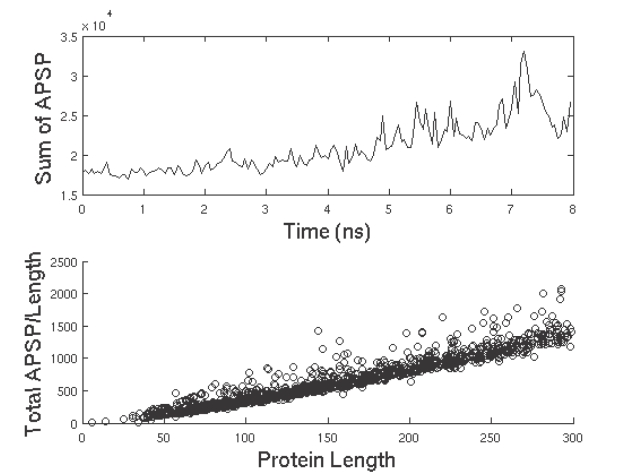
APSP over unfolding. Top panel: Plot of APSP versus the unfolding time. The APSP increases from its value at the native structure almost monotonically during unfolding. Bottom panel: Plot of APSP/Length versus protein length for 1320 single-chain proteins. The linear scaling obtained here is similar to the scaling of mean FPT/Length versus the source-target distance suggesting that there exists an optimal range of APSP values for a given protein length.

In a recent paper, del Sol et al. [28] showed that protein domains consist of modules interconnected by residues that mediate signaling through the shortest pathways. They identified these mediating residues as those that are located at inter-modular boundaries, are more rigid and tend to display a larger number of long-range than intra-modular interactions. This corresponds to the HPZ residues from our work. Since they showed that signaling in proteins, at the basis of protein allosteric behavior, is mediated through shortest paths, we calculated the shortest paths for all pairs (APSP) of residues in the protein for every given frame. Figure 12 (top panel) shows a plot of the sum of the APSP for all residue-pairs in the protein over unfolding. The APSP value tends to increase during unfolding or conversely, the APSP tends towards a minimum value during folding. Is this value of APSP a true minimum for a protein of a given length? Or, better, can we consider APSP as a useful proxy for energy minimization studies in protein folding? If this could be the case we should have a very easy to calculate descriptor for protein folding studies (see Material and Methods).

In order to answer this question, we used a dataset of 1320 single-chain proteins that we had used in an earlier work [19]. We obtained the sum of the APSP values for all residue-pairs for every protein and plotted the ratio of APSP to protein length versus the protein length as shown in the bottom panel of Figure 12. This corresponds exactly to Figure 2 of the paper by Condamin et al [22] where the ratio of mean FPT to length in scale-free networks is plotted against the source-target distance, which can be thought of as a generic measure of the ‘size’ of the system. In our case the presence of a linear relationship between APSP/Length and protein length points to the existence of a preferred range of APSP values for a given protein length possibly suggesting the existence of an optimal APSP for a given protein length. The minimization of APSP was demonstrated to be crucial for signaling proteins and regulatory linkages [28] thus furnishing a biological counterpart to the observed scaling.

### Conclusions

In this paper we build on the work by Dokholyan et al. [21] by studying the topological changes over the unfolding pathway for ubiquitin. We observed that treating a protein native structure as a network by having amino acid residues as nodes and the non-covalent interactions among them as links allows for the rationalization of many aspects of the folding process. These aspects are in line both with the already established theory of folding funnels and with the recognized importance of the FPT concept for transitions in non-homogeneous media. In protein science the application of graph theory to protein structures allowed for the elucidation of allosteric properties of proteins [23], [28]. Along these lines, we have added the possibility to uncover both foldons and ‘connector’ residues crucial for the establishment of a correct fold. Additionally, we have pointed out the importance of non-covalent interactions in protein graph connectivity, thereby indicating a sort of mechanism for the recognized role of topological properties. The possibility to derive this information directly from 3D structure opens the way to the prediction of important residues in proteins, while the confirmation of the minimization of APSP for folding allows for the establishment of a potentially useful proxy for kinetic optimality in the validation of sequence-structure predictions.

## Materials and Methods

### Molecular Dynamics Unfolding Simulations

The native structure of ubiquitin obtained from the protein data bank (PDB ID: 1UBQ) was used in the unfolding simulations carried out using NAMD [29]. Hydrogen atoms were first added to all the atoms of the structure. The structure was then checked using the WHATIF server [30]. The protein was then immersed in a water box with a using the TIP3P [31] water model with a density of 0.98 gm/cc. No additional ions were added since the overall system was charge neutral. The final system consisted of 39583 atoms with 12784 water molecules. The system was then energy minimized. CHARM22 force field and parameters [32] were used for all the calculations.

The energy minimized structure was heated to 300 K over a period of 6 pico seconds (ps). The system was then equilibrated at 300 K for 50 ps under NPT ensemble using Langevin dynamics for temperature control and Nose-Hoover Langevin piston for pressure control. The pressure was maintained at 1 atmosphere. NPT simulation was run was then performed at 300 K for 1 nanosecond (ns). SHAKE was applied to freeze the vibration of the bonds and particle mesh Ewald (PME) [33] was used to handle the long range interactions.

This was followed by heating the system from 300 K to 520 K over 22 ps after which the system was equilibrated for 50 ps using the NPT ensemble. The pressure was maintained constant at 30 bars. This was to maintain the system in an aqueous phase. The density of the system at 520 K was 0.69 gm/cc. The temperature of 520 K was selected higher than the melting temperature of ubiquitin which is 373 K at neutral pH. Finally, NVE simulations were carried out on the equilibrated system for 8 ns. Snapshots were taken once every ps.

### Global network partitioning

A brief description of the algorithm is provided. Interested readers are directed to [19] for a more detailed exposition.

We treat a protein as a network of interacting residues. Two residues in a protein are defined to interact with each other if the observed distance between any two atoms of the residues was less than the sum of their corresponding Van der Waals radii plus 1 [34] . Given a protein of length N residues an N×N adjacency matrix can then be generated that captures the network of side-chain interactions between the residues. A graph partitioning approach based on the work of Guimera et al. [35] is then used to determine the modular decomposition of a protein. The rationale for their algorithm lies in the fact that a good partition of a network must consist many intra-module links and few inter-module links. A genetic algorithm (GA) is used to maximize the modularity M defined as:
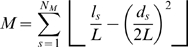
where *N_M_* is the number of modules, *L* is the number of links in the network, *l_s_* is the number of links between nodes in module *s* and *d* is the sum of the degrees of the nodes in module *s*. Since GAs are stochastic in nature, the algorithm was run 50 times on each frame and the partitioning with the highest modularity score was chosen as the “optimal” solution. Once an optimal partition of the protein into its constituent modules has been obtained, two quantities that describe the role of a given node *i* viz. the intramodule degree, and the participation coefficient are calculated. The intramodule degree of a residue measures the “connectedness” of a residue within its own module whereas the participation coefficient measures how “well distributed” the links node *i* are among the different modules. The two quantities are calculated as:
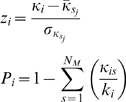
where κ*_i_* is the number of links of residue *i* to other residues in its module *s_i_*, 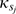 is the average of κ over all residues in module *s_j_*, and 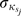 is the standard deviation of of κ in module *s_j_*, κ*_is_* is the number of links of node *i* to nodes in module *s* and *k_i_* is the total degree of node *i*. The readers are referred to Guimera et al [35] for a full exposition of the meaning of the participation coefficient and the intra-module degree.

### Recurrence Quantification Analysis (RQA)

RQA is a nonlinear analysis technique developed by Webber and Zbilut [36] and has been applied to diverse fields [36]–[39]. RQA is based on the concept of recurrences which is simply a point which repeats itself. Unlike other methods such as Fourier transforms or wavelets, RQA requires no transformation of data and can be used for both linear and nonlinear systems. Given a reference point *X*_0_, a point *X* is said to recur with reference to *X*_0_ if
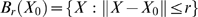
where *B_r_* is a ball of radius *r*. Here a recurrence plot of a simulated sequence is reported so to give a direct appreciation of the meaning of RQA parameters:

REC is given as:
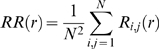
where *R_i,j_* is the recurrence between the *i^th^* and *j^th^* vectors.

DET is given as
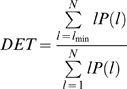
where *l* is the line length and *P*(*l*) is the line length histogram. Finally, Shannon's entropy is defined as
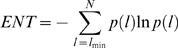
where *p*(*l*) = *P*(*l*)/*N_l_* and 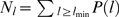. *P*(*l*) is the total number of diagonal lines.

### Topological Parameters

The all-pairs shortest paths (APSP) aims to compute the shortest path from each node *i* to every other node *j*. The APSP for all the nodes of the protein network were obtained using the Floyd-Warshall algorithm [40].

The closeness centrality for a given residue is defined as:
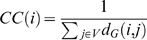
where *d_G_*(*i*,*j*) is the geodesic distance (shortest path) between residues *i* and *j*.

### Projection of all-pairs RMSD distance onto 3D Cartesian space

The projection of the pairwise RMSD between all pairs of frames (one every 50 ps) was carried out by minimizing the Euclidean distance between the actual pairwise distance and the distance in the 3D space. The function to be minimized is given by [25]:
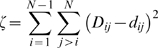
where *D_ij_* is the RMSD between the i^th^ and the *j^th^* structures and *d_ij_*, the distance in the 3D Cartesian space is given by
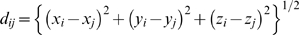
The function ζ was minimized using a genetic algorithm (GA) to obtain the coordinates in the Cartesian space that would best represent the inter-frame RMSDs.
